# Accuracy of RNAseq based SNP discovery and genotyping in *Populusnigra*

**DOI:** 10.1186/s12864-018-5239-z

**Published:** 2018-12-12

**Authors:** Odile Rogier, Aurélien Chateigner, Souhila Amanzougarene, Marie-Claude Lesage-Descauses, Sandrine Balzergue, Véronique Brunaud, José Caius, Ludivine Soubigou-Taconnat, Véronique Jorge, Vincent Segura

**Affiliations:** 1 BioForA, INRA, ONF, Orléans, 45075 France; 2 Institute of Plant Sciences Paris-Saclay (IPS2), CNRS, INRA, Université Paris-Sud, Université Paris-Saclay, Université d’Evry, Université Paris-Diderot, Sorbonne Paris-Cité, Orsay, 91405 France; 3 IRHS, INRA, Agrocampus-Ouest, Université d’Angers, SFR 4207 QUASAV, Beaucouzé, 49071 France

**Keywords:** DNA polymorphisms, Bioinformatics pipeline, Black poplar, Transcriptomics

## Abstract

**Backgroud:**

*Populus nigra* is a major tree species of ecological and economic importance for which several initiatives have been set up to create genomic resources. In order to access the large number of Single Nucleotide Polymorphisms (SNPs) typically needed to carry out a genome scan, the present study aimed at evaluating RNA sequencing as a tool to discover and type SNPs in genes within natural populations of *P. nigra*.

**Results:**

We have devised a bioinformatics pipeline to call and type SNPs from RNAseq reads and applied it to *P. nigra* transcriptomic data. The accuracy of the resulting RNAseq-based SNP calling and typing has been evaluated by (i) comparing their position and alleles to those previously reported in candidate genes, (ii) assessing their genotyping accuracy with respect to a previously available SNP chip and (iii) evaluating their inter-annual repeatability. We found that a combination of several callers yields a good compromise between the number of variants type and the accuracy of genotyping. We further used the resulting genotypic data to carry out basic genetic analyses whose results confirm the quality of the RNAseq-based SNP dataset.

**Conclusions:**

We demonstrated the potential and accuracy of RNAseq as an efficient way to genotype SNPs in *P. nigra*. These results open prospects towards the use of this technology for quantitative and population genomics studies.

**Electronic supplementary material:**

The online version of this article (10.1186/s12864-018-5239-z) contains supplementary material, which is available to authorized users.

## Background

*Populus nigra* is a major tree species from Eurasian riparian ecosystems and one of the 3 main parental species used in poplar breeding programs to develop highly productive interspecific cultivated hybrids. For these reasons, several initiatives have recently been set up to create genomic resources within this species as tools to improve conservation and breeding strategies [1, 2]. The main objective of such initiatives is to discover and type genomic variants like Single Nucleotide Polymorphisms (SNPs) for various applications, including the identification and quantification of introgressions from the cultivated compartment, the study of population structure and the identification of variants associated with economically or ecologically relevant phenotypes through association genetics.

Early studies in *P. nigra* have focused on re-sequencing specific candidate genes from the lignin pathway [3–5], but more recent work has broadened the scope of analyses through the development of a genotyping chip from SNPs detected by whole-genome sequencing [1, 2]. This genotyping tool was successfully used to study the structure of the genetic diversity of the species [1] and to identify some genomic regions associated with economically important traits [6]. However, the genotyping was limited to 7903 SNPs preferentially located within particular candidate regions underlying some Quantitative Trait Loci (QTLs) previously reported in biparental crosses. Moreover, the frequency of the SNPs within *P. nigra* populations appeared to be upwardly biased, limiting the analyses to common variants [1]. Consequently, the application of this chip especially in association genetics could be limited as underlined by the low number of significant associations reported [6]. Indeed, given the rapid Linkage Disequilibrium (LD) decay within this species and its genome size, an exhaustive genome-wide association study (GWAS) would require between 67,000 and 134,000 evenly spaced SNPs which is between 8 and 16 times more than the number of SNPs available from the chip cited above [7, 8].

In order to access a large number of SNPs, as typically needed for an exhaustive GWAS in *P. nigra*, several options relying on next-generation sequencing would be available. If whole genome sequencing appears to be still too expensive for a fairly large number of genotypes, reducing the complexity of the genome prior to sequencing for instance with restriction enzymes (GBS [9]; RADseq [10]), or sequence capture (exome sequencing, [11]) seems to be a promising way forward for reaching the objectives. Indeed, sequence capture has recently successfully been used to genotype around 350,000 SNPs in *P. deltoides* and identify putative regulators of bioenergy traits [12]. RNA sequencing (RNAseq) represents also a cost-effective way to reduce complexity while focusing on the expressed fraction of the genome [13]. However, to date, RNAseq has more often been used for SNP discovery than for direct genotyping of large populations. For instance, Geraldes et al. [14] found around 500,000 SNPs through RNAseq of developing secondary xylem in *P. trichocarpa*, and later on, a SNP chip was developed partly from the previously discovered RNAseq SNPs [8] in order to further carry out association scans [15, 16]. Nevertheless, recent studies have been using RNAseq as a tool for both discovering and genotyping a large number of SNPs in populations [17–21], underlining the interest of this approach for population and quantitative genomics studies. However, to our knowledge, no study so far has evaluated the accuracy of SNP genotyping from RNAseq data.

The present study aims at evaluating RNAseq as a tool to type a sufficiently large amount of SNPs within natural populations of *P. nigra* to carry out a GWAS. For that purpose, we performed RNAseq on pools of young differentiated xylem and cambium collected on 2 biological replicates of 12 genotypes originated from 6 natural populations. We have further developed a dedicated bioinformatic pipeline to discover and type SNPs within the sequences. The accuracy of the resulting RNAseq-based SNPs has also been evaluated by (i) comparing their position and alleles to those previously reported in candidate genes [3, 4], (ii) assessing their genotyping accuracy with respect to a SNP chip [1], (iii) evaluating their interannual repeatability. Finally, the resulting validated SNPs have been used to perform basic genetic analyses to illustrate the usefulness of the released SNP dataset.

## Methods

### Plant material, experimental design and tissue sampling

Trees were sampled in an experimental site established in a common garden in 2008 in Central France (Orléans, Loire Valley, 47°50’N 01°54’E, 108 m above sea level) at INRA. The experimental site is described in Guet et al. [22]. Briefly, a *P. nigra* collection composed of 1098 cloned genotypes sampled in natural populations present in 11 river catchments in four European countries was planted according to a randomized complete block design with a single tree per block and six replicates per genotype. The trees have been growing through three short rotations since the planting, they were cut back in March 2010 and in February 2012. The experiment was carried out in accordance with local legislation.

Twelve genotypes belonging to 6 river catchments (as defined by Guet et al. [22]: Adour, Dranse, Loire, Ramières, Rhin, Ticino) were selected for the present study to represent the range of available geographical origin in France and Northern Italy. The genotypes from the French populations (Adour: BDX-003, AST-005; Dranse: DRA-045, DRA-038; Loire: VDL-018, 92510-1; Ramières: 1-J31, 1-A26; and Rhin: STR-010, RHN-028) were collected and are owned by INRA (UMR0588-BioForA), while those from Italy (Ticino: SN-2, SN-7) are owned and were kindly provided by the University of Tuscia. Two trees per genotype were sampled in June 2014 (in blocks 2 and 4). The most vigorous stem of each tree was cut back and the bark was detached from the trunk in order to scratch young differentiating xylem and cambium tissues using a scalpel. The tissues were immediately immersed in liquid nitrogen and crudely ground before storage at -80°C pending the RNA extraction.

### RNA extraction, library preparation and sequencing

For each biological repetition and each tissue, samples of young differentiating xylem and cambium were ground with a swing mill (Retsch, Germany) and tungstene beads under cryogenic conditions with liquid nitrogen during 25 s (frequency 25 cps/s). Powders were stored at -80°C until RNA extraction. About 100 mg of ground tissue was used to isolate separately total RNA from xylem and cambium of each plant with RNeasy Plant kit (Qiagen, France) according to manufacturer’s recommendations. Treatment with DNase I (Qiagen, France) to ensure elimination of genomic DNA was made during this purification step. RNA was eluted in RNAse-DNAse free water and quantified with a Nanodrop spectrophotometer. RNA from xylem and cambium of the same plant were pooled in an equimolar extract (250 ng/ *μ*L) before being sent to the sequencing platform.

RNAseq experiment was carried out at the platform POPS (transcriptOmic Platform of Institute of Plant Sciences - Paris-Saclay) thanks to IG-CNS Illumina Hiseq2000. RNAseq libraries were prepared from polyA RNA selection using TruSeq_Stranded_mRNA_SamplePrep_Guide_15031047_D protocol (Illumina$\circledR $, California, U.S.A.). Eight libraries were multiplexed per lane and paired-end (PE) sequenced on an Illumina HiSeq2000. Thus, over 22 million of 100 base pairs (bp) PE-reads were generated per sample.

### Sequencing data processing and variant calling pipeline

**Fig. 1:**
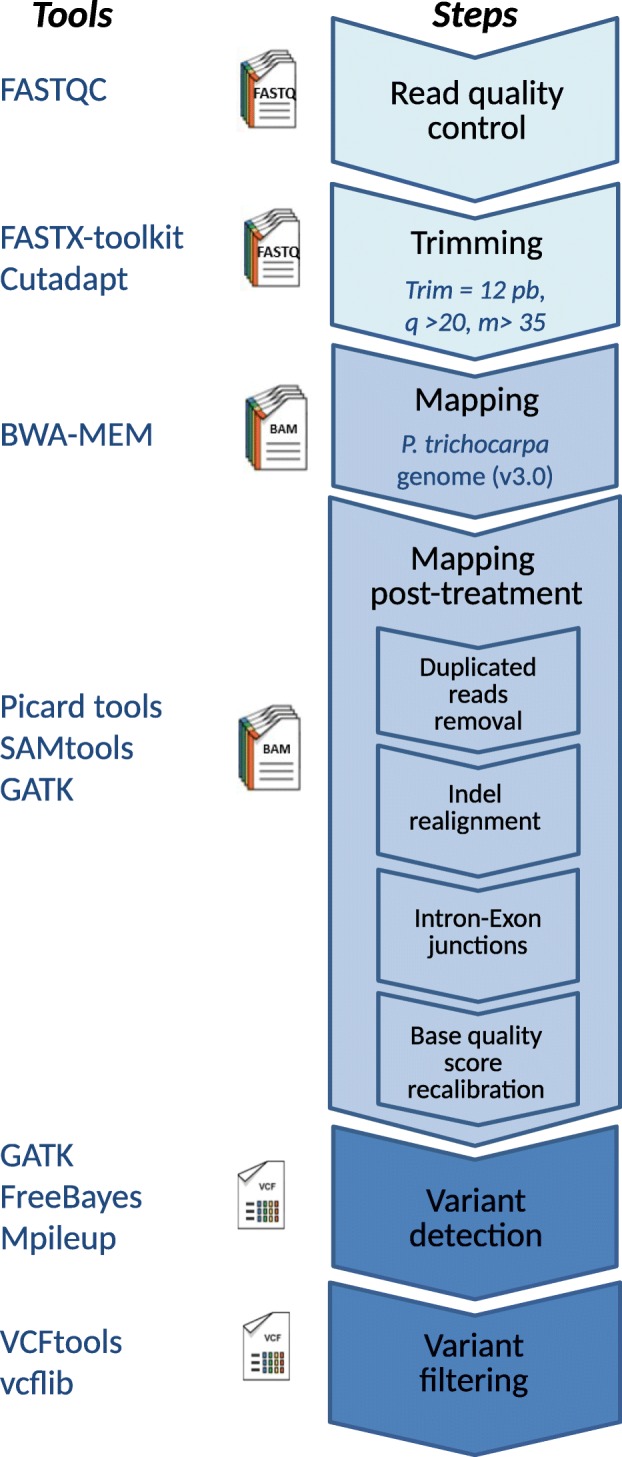
Variant calling pipeline. Schematic representation of the bioinformatic pipeline devised for variant calling and genotyping from mRNA sequencing reads. References and parameters for each tools are indicated in the method section

We have devised a bioinformatic pipeline for processing the reads, mapping them to a reference genome and calling the SNPs using several callers (Fig. 1). Each step of this pipeline is described hereafter.

Read quality control was assessed using FastQC (v0.11.4; [23]). Cutadapt 1.10 [24] and the FASTX-toolkit 0.0.13 [25] were used to remove adaptor sequences and low-quality bases. The 13 first 5’ bases were removed, as well as bases with PHRED score below 20 from the 3’ end of the read. Only reads longer than 35 nucleotides were kept.

Reads were aligned to the *Populus trichocarpa* reference genome version 3.0, retrieved from the JGI Comparative Plant Genomics Portal [26, 27]. Alignment was performed using the short read aligner BWA-MEM 0.7.12 [28] with default parameters using the paired-ends information to produce per-tree SAM files that were converted to BAM files and sorted by aligned position on the reference with SAMtools 1.3 [29]. As an alternative we also tested TopHat [30], but BWA-MEM with default settings yielded the highest percentage of mapping and was thus selected.

The data pre-processing steps recommended in the GATK best practices workflow [31, 32] were performed before variant identification. PCR duplicates were marked with the MarkDuplicates from Picard tools 2.0.1 utility [33] to mitigate biases introduced by data generation steps such as PCR amplification or minimize gene expression variations. We also performed local realignment around indels, checked intron-exon junctions and recalibrated the base quality scores with GATK 3.1 [34].

Four different variant callers were used to perform SNP and indel discovery and genotyping across all 24 samples simultaneously (Table 1): (i) GATK 3.1 [31, 32, 34] using the HaplotypeCaller tool in multi-sample calling mode (modality “GATK”); (ii) GATK 3.1 using the HaplotypeCaller tool in single-sample calling mode followed by joint genotyping of the samples with the GenotypeGVCFs tool (modality “gVCF_GATK”); (iii) FreeBayes 0.9.20 [35] in multi-sample calling mode (modality “FreeBayes”); and (iv) the mpileup command from SAMtools 1.3 [29] in multi-sample calling mode followed by bcftools 1.3.1 [36] with the multiallelic calling model (modality “Mpileup”). Default parameters were used.

**Table 1:** Number of variants detected for each of the 7 calling modalities tested in the present study

Variant calling modality	Missing value in genotype calls		
	noNA	2NA	anyNA
GATK	464,829	555,828	902,256
gVCF_GATK	407,037	497,314	927,522
FreeBayes	492,073	640,445	795,459
Mpileup	496,688	594,932	949,411
3Callers	341,584	366,123	400,392
4Callers	252,887	262,447	271,399
3CallersConsensus	356,275	442,931	785,377

“noNA”: no missing value; “2NA”: lower or equal to 2 missing values; “anyNA”: no filter on missing values. “3Callers”: SNPs detected with at least 3 callers; “4Callers”: SNPs detected with 4 callers; “3CallersConsensus”: SNPs detected with at least 3 callers with correction of the genotype calling when discrepancies existed between callers (see details in Methods)

We obtained 4 files in a raw Variant Call Format (VCF) with no filter. The functions vcfallelicprimitives and vcfcreatemulti from vcflib [37] were used to decompose the complex variants generated by FreeBayes into a canonical SNP and indel representation. For each caller individually, severals filtering parameters were applied with VCFtools 0.1.15 [38]: selection of biallelic SNPs (indels and not-biallelic SNPs were removed); SNP quality threshold ≥ 30; intra-specific polymorphisms (*P. nigra*).

In order to generate a high-confidence SNP set, the SNPs identified by 3 or 4 callers were selected using the vcf-isec tool from VCFtools 0.1.15. We first considered positions with the same genotype across all individuals with 3 or 4 callers (modalities “3Callers” and “4Callers”, Table 1). Because some SNPs could display a difference between callers for only a limited number of individuals, we further considered a consensus set between 3 callers (modality “3CallersConsensus”, Table 1). In this case, for a given individual, when at least 3 callers agreed, the resulting genotype call was set as the consensus between them, otherwise the genotype call was set as a missing value for this particular individual. This part was done using home-made scripts.

In the end, we considered 7 modalities for which we tested 3 different filters for missing values in the genotype calls (Table 1): no missing value allowed (“noNA”), up to 2 missing values allowed (“2NA”) and any missing value allowed (“anyNA”).

### Validation of the SNPs detected and genotype calls

A first validation of the SNPs detected and genotyped by each or by combinations of the callers has been done through a comparison of the genotype calls with those previously obtained with a 12k Illumina Infinium BeadChip array [1]. Full details of SNP discovery and selection, array development and data filtering criteria are given in Faivre-Rampant et al. [1]. In brief, 852 unrelated *P. nigra* accessions (including our 12 genotypes) were successfully genotyped with this genotyping array, yielding 7903 SNPs for the validation of genotype calls. For each of the 12 genotypes, genotyping accuracy was calculated as the percentage of similarity between chip genotype and RNAseq genotype at the common positions.

A second validation consisted in comparing the SNPs detected with those previously identified within 5 candidate genes through Sanger and Next-Generation sequencing (CAD4, HCT1, C3H3, CCR7, and 4CL3; [3, 4]). The originally reported SNP were repositioned by aligning reference sequences with the latest *P. trichocarpa* reference genome assembly (v3.0; [26, 27]; Additional file 1).

A third validation consisted in evaluating an inter-annual repeatability of the RNAseq genotype calls by conducting the same experiment on other ramets of the same 12 genotypes one year later. Two other ramets of each genotype were sampled in June 2015 (in blocks 1 and 3 of the same experimental design). The RNA extraction and library preparation were the same as described above. The RNAseq samples have been sequenced in Single-Read (SR) in this second experiment, multiplexing ten samples per lane. This setup yielded approximately 20 millions of SRs per sample. The same bioinformatic pipeline was used on this data, except for the mapping step where we accounted for the single nature of the reads.

The usefulness and relevance of the resulting SNPs for basic genetic studies were further assessed as another form of validation. Minor Allele Frequency (MAF) was calculated with VCFtools 0.1.15 [38]. Genome-wide distribution of SNPs was calculated based on a 100-kb window with custom R scripts [39]. SNP density within a 100-kb window was further correlated with the sum of the expression of the genes located in the same window. The SNPs have also been annotated using Annovar (version 2017Jul16) [40]. We further tested whether the gene models (with at least 5 SNPs) displayed any enrichment in Gene Ontology (GO) terms using *Arabidopsis thaliana* annotation with the R package topReviGO [41]. Finally, population structure was described using a hierarchical ascendant clustering on a distance matrix estimated as *d*=1−*IBS*, where IBS is the identity by state matrix between genotypes computed with PLINK 1.07 [42].

## Results

### Quality control, mapping and post-treatment

The trimming process removed 0.3% of reads and only 7% of duplicated reads were rejected. At the mapping step, around 99.7% of the reads were mapped against the reference genome (*P. trichocarpa*) and 93.3% were mapped without ambiguous position, even with RNA extracts from a different species (*P. nigra*). A first crude SNP detection and calling on each of the 24 samples with a single caller (“FreeBayes”) enabled the identification of between 772,043 and 1,156,297 SNPs depending on the sample, of which some were previously genotyped on the same individuals with a SNP array [1]. These common SNPs were used to compare the genotype calls and further identified that 3 of the 24 samples did not match perfectly the original genotypes (genotyping accuracy less than 90%, Additional file 3: Figure S1). These three samples corresponded to one repetition of the 3 genotypes “1-A26”, “RHN-28”, and “STR-10”. They were removed from further analyses. The remaining 21 mapping BAM files were used for SNP detection and genotyping at the genotype level (using genotype as a read group). In other terms, for 9 out of 12 genotypes we used reads from two samples, increasing the sequencing depth available. Validation of genotype identity has been made afterwards using the same SNP array as previously (Additional file 3: Figure S2).

### SNP detection and genotyping in 12 genotypes

**Fig. 2:**
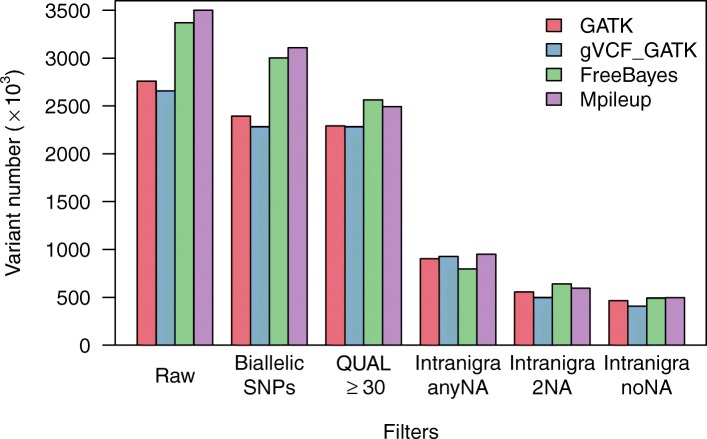
Variant discovery in 12 *Populus nigra* genotypes. Number of variants discovered with each of the four callers (“GATK” in red; “gVCF_GATK” in blue; “FreeBayes” in green and “Mpileup” in purple) after applying different filters (“Raw”: no filters; “Biallelic SNPs”: indel removed; only biallelic SNPs retained; “QUAL ≥ 30”: SNP quality greater than 30 retained; “Intranigra/anyNA”: SNP polymorphic in *P. nigra* retained; “Intranigra/2NA”: SNP polymorphic in *P. nigra* with at most 2 missing genotype values retained; “noNA”: SNP polymorphic in *P. nigra* without missing genotype value retained)

Between 2,658,024 variants (included intra- and interspecific SNPs and Indels) for “gVCF_GATK” and 3,500,381 variants for “Mpileup” were detected depending on the caller used (Fig. 2; Additional file 2: Table S1). Among filters applied, the selection of *P. nigr*a intra-specific SNPs was the criteria that reduced most drastically the numbers of detected SNPs (from 795,459 SNPs for “FreeBayes” to 949,411 SNPs for “Mpileup”). The final number of SNPs detected without missing genotype was fairly similar for all callers, ranging between 407,037 SNPs for “gVCF_GATK” to 496,688 for “Mpileup”.

We further compared the *P. nigra* SNP positions and genotype calls between each callers as well as combinations of at least two callers (Table 1). As expected the number of SNPs detected was lower when considering combinations of callers rather than single ones. Indeed, the “core” SNP set detected by all callers contained 252,887 SNPs with no missing genotype calls (“4Callers-noNA”). This number increased to 341,584 SNPs with no missing genotypes when considering at least 3 callers (“3Callers-noNA”). A further increase could be obtained when computing a consensus genotypes between the callers (“3CallersConsensus-noNA”, 356,275 SNPs) but this gain was much more pronounced when allowing missing genotypes calls (“3CallersConsensus-2NA”, 442,931 SNPs; “3CallersConsensus-anyNA”, 785,377 SNPs), underlining the interest of computing a consensus genotyping when combining multiple callers.

### SNP validation

**Fig. 3:**
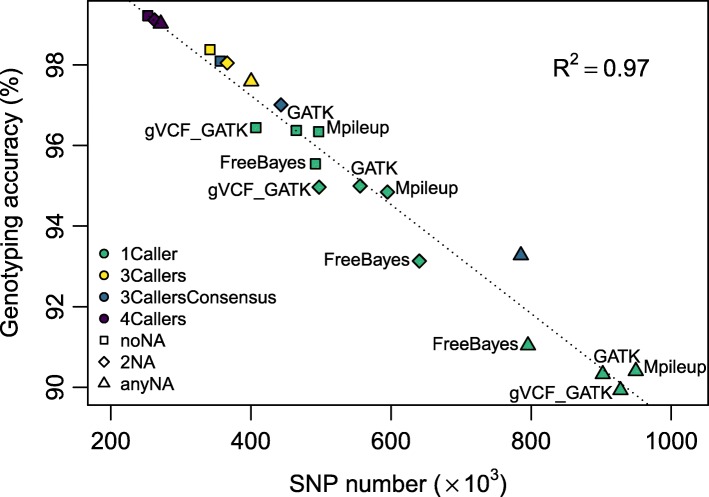
Relationship between genotyping accuracy and the number of SNPs detected. Number of SNPs detected and genotyping accuracy for 7 calling modalities times 3 options for missing values. See Table 1 for the corresponding denominations

A total of 7903 SNPs previously genotyped with a SNP array [1] were compared with the list of SNPs detected with each caller, combination of 3 or 4 callers (Fig. 3; Additional file 2: Table S2; Additional file 3: Figure S3). Genotyping accuracy, evaluated as the percentage of similarity over all common positions, varied from 90 to 99% and was negatively correlated with the total number of SNP detected and consequently the number of positions available for the comparison. Thus, there is a trade-off between the number of SNPs we are willing to obtain and the quality of the genotyping information. The negative relationship between the number of SNPs detected and the genotyping accuracy appeared to be linear (*R*^2^=0.97).

The position of the calling methods with respect to the regression line provides information on their performance for variant detection and genotyping. “FreeBayes” and “gVCF_GATK” were always below the line and thus appeared to be the less accurate with respect to the number of variants detected. “GATK” and the combination of 4 callers were always very close to the line and thus could be seen as intermediary performing calling methods. Finally, “Mpileup” and the combination of 3 callers were always above the line, suggesting that they performed best. Of note “3CallersConsensus-anyNA” was the most distant modality above the line, underlining the strength of this approach for detecting and genotyping variants in our dataset.

**Fig. 4:**
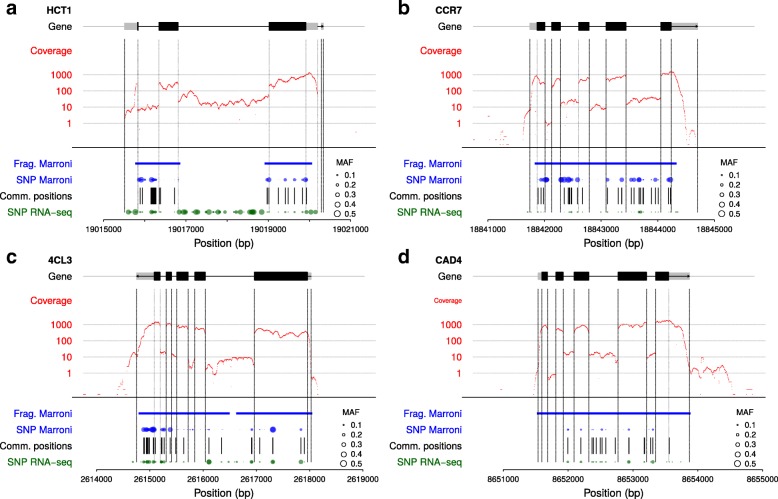
Positions of SNPs discovered and genotyped with RNAseq across 12 *Populus nigra* individuals and along four genes. **a**. “HCT1” (Potri.003G183900); **b**. “CCR7” (Potri.003G181400); **c**. “4CL3” (Potri.001G036900); **d**. “CAD4” (Potri.009G095800). “Coverage” in red refers to the mean depth at each position among the 12 individuals; “Frag. Marroni” and “SNP Marroni” in blue refer to sequenced fragments and SNPs discovered and typed in [3, 4]; “SNP RNA-seq” in green refers to SNPs discovered and typed in the present study with the modality “3CallersConsensus-noNA”. Point symbol size for each SNP is proportional to its MAF across the 12 individuals

For further analyses and validations, we decided to focus on the set of SNPs that gave the highest number of SNPs with at least 98% of accuracy, *i.e.* the consensus from 3 callers with no missing data (modality “3CallersConsensus-noNA”). The resulting 356,275 SNP positions were further compared to previously reported *P. nigra* SNPs obtained by Sanger or NGS sequencing of five candidate genes fragments, which were also used to compare detected SNP positions [3, 4] (Fig. 4; Additional file 3: Figure S4). Because these candidate genes were expressed within our samples, a fairly large amount of previously identified SNPs were also detected in our study even within introns. Indeed, the number of positions also detected with RNAseq varied between 30 to 61%. It is worth noting that a fairly large number of SNPs were detected in introns in these candidate genes (“HCT” : 53/70; “4CL3”: 15/36; “C3H3”: 24/45; “CAD4”: 10/19; “CCR7”: 20/39). Another gene was analyzed in detail because it included a large number of SNPs that have been genotyped with the SNP array (Potri.017G084100): 9 from the 19 SNPs used in the array were detected with RNAseq (Additional file 3: Figure S4).

Finally, we carried out an inter-annual repeatability analysis of the genotyping by RNA sequencing approach. Because the second sequencing experiment was done in a single read setting, we detected around twice less positions than in the first experiment (157,569 *vs.* 356,275 with the “3CallersConsensus-noNA” modality). Of note, 88% of the SNPs detected in the second experiment were also found at the same position with the same genotype calls in the first experiment.

### SNP characterization and usefulness

**Fig. 5:**
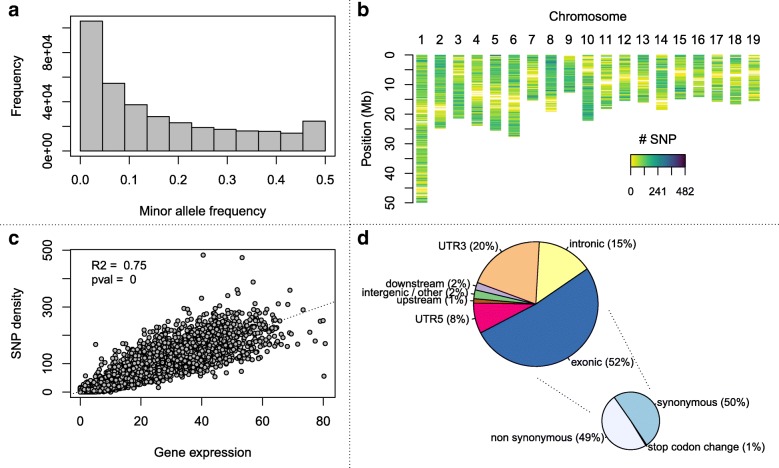
Description of the final set of discovered SNPs. **a** Minor Allele Frequency distribution; **b**. Density of SNPs across the genome (100kb windows); **c**. Relationship between SNP density and gene expression; **d**. Annotation of SNPs

We estimated the minor allele frequency for each of the 356,275 SNPs from the modality “3CallersConsensus-noNA”. The distribution had an L-shape with an excess of rare alleles as expected under population genetics models (Fig. 5a).

To evaluate the genomic distribution of our SNPs, we computed the density within 100-kb windows of the 351,157 SNPs located on the 19 chromosomes of *P. trichocarpa* v3.0. The number of SNPs within 100-kb windows ranged between 0 and 482 with an average of 89 and a median of 83. Moreover, 92% of the 100-kb windows harbored at least 1 SNP, underlining an overall good coverage of the genome (Fig. 5b). To further explain the observed variations in SNP density within 100-kb windows, we compared this numbers to the sum of gene expressions within the same windows, estimated as the log2 of read counts per million. We found a highly significant positive relationship between gene expression and the number of SNPs detected (*R*^2^=0.75, Fig. 5c).

The automatic annotation of our SNPs highlighted as expected that the vast majority of them (80%) were located within exons or 3’ and 5’ UTRs (Fig. 5d). Nevertheless, as already observed when comparing with SNPs previously reported within candidate genes, a fairly large amount of SNPs were located within introns (15%, Fig. 5d). These intronic SNPs are likely to come from pre-mRNA [43]. Considering exonic SNPs, their annotation highlighted a very low number of mutations affecting the stop codon (1%). The remaining exonic SNPs were almost equally split between synonymous and nonsynonymous sites (Fig. 5d).

In the end, we found that 19,249 genes were covered by at least 5 SNPs which corresponds to 47% of gene models in *P. trichocarpa* genome annotation. We further tested whether these 19,249 gene models displayed any enrichment in GO terms using *Arabidopsis thaliana* annotation (18,384 orthologs). We found that few GO terms were enriched within our set, but they corresponded to biological processes that seem to be quite generic rather than specific to the tissues sampled (Additional file 3: Figure S5).

**Fig. 6:**
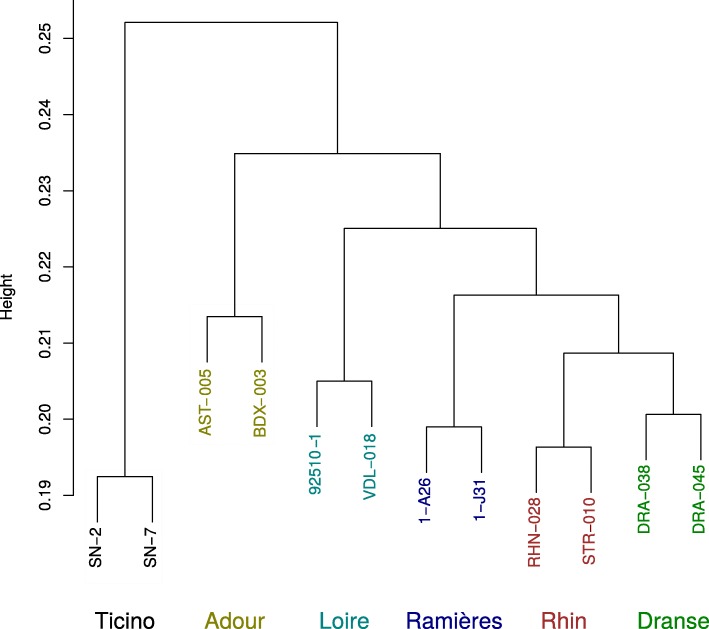
Hierarchical ascendant clustering of 12 *Populus nigra* individuals. The analysis was carried out with 250,784 SNPs from the “3CallersConsensus” modality after filtering SNPs with a minor allele frequency below 0.05

Finally, we used the 250,784 SNPs with a MAF higher than 5% to evaluate the genetic structure of our 12 genotypes (Fig. 6). A hierarchical ascendant clustering of the genotypes clearly highlighted 6 groups corresponding to the populations to which the genotypes belong to. It is worth mentioning that the population clustering matched their geographic origins.

## Discussion

We have successfully built a pipeline from multiple bioinformatics tools for detecting and typing several hundred thousand SNPs from RNAseq data. Genotyping accuracy of the resulting SNPs has been evaluated by (i) a comparison with genotyping data previously obtained with a SNP array [1] and (ii) an interannual validation. The high accuracy (around 95%) underlined the quality of the genotypic dataset obtained with our pipeline. Additionally, when looking at candidate genes for wood properties (lignin pathway), many SNPs previously reported by DNA sequencing could be recovered within our RNAseq data even if our study focused only on 12 genotypes. This could be expected because 3 and 7 of our 12 genotypes were also included in previous sequencing studies [3, 4]. The resulting variants frequency spectrum followed the expectations from population genetics models and they were spread across most of the genome. The very few genomic regions that appeared to be uncovered correspond to predicted centromeric regions [2] which do not carry many gene models and thus cannot be tagged in an RNAseq experiment.

If the vast majority of SNPs were as expected located within exonic or UTR regions, it is interesting to note that a fairly large number of SNPs appeared to locate within introns. Several hypotheses could explain this result. First, we have used as a reference the genomic annotation from a different species within the same genus: *P. trichocarpa*. If most of our reads mapped to this reference genome, interspecific variability is likely to have affected the quality of the annotation of our SNPs. In addition, alternative splicing has been shown to be frequent in developing xylem of *P. trichocarpa* [44]. This phenomenon is likely to be more frequent at the interspecific level and may thus have contributed here to the intronic SNPs detected. Second, if in RNAseq most of the reads come from mature mRNA, it has been shown that pre-RNA could as well be sequenced which would yield reads outside of the exonic and UTR regions [43]. Actually, the read coverage was not null in the introns of our candidate genes, providing sufficient information for SNP detection and typing. Thus, it was not surprising to have intronic SNPs within RNAseq data, especially for highly expressed genes as expected here for candidate genes from the lignin pathways, since we sampled our RNA from young differentiating xylem and cambium. Indeed, we have also found a highly significant positive correlation between gene expression level and SNP density, but this observation did not necessarily pop up when we observed the SNPs detected, their frequency and the read coverage on the candidate genes from the lignin pathway. As a matter of fact, the frequency of SNPs did not seem to vary a lot between highly covered exonic regions and weakly covered intronic regions. For “HCT1” (Potri.003G183900), the frequency of SNPs even seems to be higher in introns than exons. This is consistent with the negative Tajima’s D previously obtained on the set of candidate genes from the lignin pathway in *P. nigra* [3, 4] as well as for genes associated with a lower lignin content in *P. deltoides* [12]. In addition, Marroni et al. [3] also reported for “HCT1” a non-synonymous to synonymous nucleotide diversity ratio of 0.03 suggesting that this gene is under purifying selection which may explain the pattern observed here. Consequently, the frequency spectrum of SNPs from RNAseq reads is likely to be complex as both affected by gene expression levels as well as evolutionary factors. Care must thus be taken when using these data for population genomic analyses and especially for detecting signatures of selection.

We used several variant callers as well as their combination. Given the observed and expected trade-off between genotyping accuracy and the number of SNPs detected, we found that this strategy was efficient since better performances could be reached through the combination of multiple callers rather than using a single one, except for Mpileup. The gain was mainly due to the production of consensus genotype calls from the different callers, especially when they did not all agree. This opens the choice between various options along the accuracy amount trade-off, which could further be picked depending on the objectives of downstream analyses. For instance, if one wishes to obtain the largest number of SNPs within its dataset for carrying out a GWAS, it may be a good idea to use the combination of 3 callers with missing data allowed and then to impute the missing data with a dedicated tool which make use of linkage disequilibrium between neighbouring SNPs for the imputation [45]. If one wants to only use 1 caller, we recommend the use of “Mpileup”, as it is the only one that produced a data set at the equilibrium between quantity and quality of SNPs. Finally, if one wants to have the best quality of SNPs at the price of a lower number, we recommend the use of the 4 callers data intersection, without missing data.

In the present work, we have focused on biallelic SNPs because they constitute the most abundant polymorphism in the genome. However, the callers used have also detected numerous indels or triallelic SNPs which could prove useful for various analyses and thus would deserve further work. Also, because we used a different species as a reference for mapping our reads and annotating our SNPs, a large amount of the SNPs detected by each of the callers displayed interspecific variation as underlined by the steep decrease in the number of variants when considering intra-nigra polymorphism only. These polymorphisms could also be valuable for species determination and for studying interspecific hybridization [46].

We sampled our RNA from two tissues, young differentiating xylem and cambium, because our research focuses on wood production. Combining information from two tissues has likely increased the number of genes covered by reads and consequently by SNPs compared to what would have been obtained when considering a single tissue. Using this strategy, we could obtain a genotyping dataset with almost half of the gene models of *P. trichocarpa* covered by at least 5 SNPs. Morevover, the GO enrichment analysis suggested that sampling did not introduce a strong bias into the representativeness of functional categories that were effectively captured by the RNAseq experiment. One strategy to increase the genomic coverage could be to combine RNA from multiple tissues but this would have a cost in term of sequencing. More generally, because many factors affect gene expression such as the developmental stage or the tissue considered, further works are required to assess how this impacts genotyping with RNAseq.

## Conclusion

In order to identify loci which matter for explaining quantitative trait variation or involved in adaptation to biotic or abiotic constraints, one needs to investigate a large number of individuals to reach a sufficient statistical power. But for a given amount of money to be spent in a sequencing experiment, there is a tradeoff between the sample size and the extent of the genome that can be examined [47]. Several methods have been proposed to reduce the complexity of the genome prior to sequencing enabling the multiplexing of individuals onto a sequencer lane. Here we have used RNAseq as a ’natural’ alternative to reduce genome complexity prior to sequencing and have shown with several validations that it is efficient for the simultaneous discovery and typing of SNP. If all of these genome complexity reduction techniques have pros and cons [48–51], we believe that RNAseq has far been underexploited by comparison to the others and hope that our results will encourage its future use. One reason for the unpopularity of RNAseq for genotyping might be its cost which remains fairly expensive in comparison to GBS or RADseq, but one should also note that it also enables the access to the expression of genes in the tissue sampled which together with the SNPs generated can be used to detect eQTLs or ASE [13, 52].

## Additional files

Additional file 1 Position of SNPs on *Populus trichocarpa v3.0 genome version of the SNP identified by Marroni et al. [*3*,*^4^*] on 5 genes.* (XLSX 157 kb)

Additional file 2 Number of SNPs detected by different callers or combinations of callers and using different filters. **Table S1**: Total number of SNPs detected with four different callers and applying different filters. **Table S2**: Comparison of number of SNPs detected with RNAseq data, identical positions with the SNP chip and genotyping accuracy using 7 calling modalities times 3 options for missing values. (XLSX 12 kb)

Additional file 3 **Figure S2**: Distribution of genotyping accuracy of RNAseq data computed from a comparison with genotyping from a previously available SNP array [1] for the 12 individuals used in the study. **Figure S3**: Variation of the total SNP number and identical positions found with the chip data using 7 calling modalities times 3 options for missing values. **Figure S4**: Positions of SNPs discovered and genotyped with RNAseq across 12 *Populus nigra* individuals and along two genes. **Figure S5**: Graphical representation of the enrichment in GO terms (biological process) for the genes covered by at least 5 SNPs. (PDF 434 kb)

## Abbreviations

ASE: Allele-specific expression
BAM: Binary alignment map
BP: Base pair
BWA: Burrows-wheeler aligner
DNA: DeoxyriboNucleic acid
DNase: DeoxyriboNuclease
eQTL: expression quantitative trait locus
GATK: Genome analysis ToolKit
GBS: Genotyping by sequencing
GO: Gene ontology
GWAS: Genome-wide association study
LD: Linkage disequilibrium
MAF: Minor allele frequency
PCA: Principal component analysis
PCR: Polymerase chain reaction
PE: Paired-end
QTL: Quantitative trait loci
RAD: Restriction site associated DNA
RADseq: RAD sequencing
RNA: RiboNucleic acid
RNase: Ribonuclease
RNAseq: RNA sequencing
SAM: Sequence alignment map
SNP: Single nucleotide polymorphism
SR: Single-read
UTR: UnTranslated region
VCF: Variant call format
