# Supplementary material for: Accuracy of RNAseq based SNP discovery and genotyping in Populusnigra
Source: BMC Genomics. 2018 Dec 12;19:909. doi: 10.1186/s12864-018-5239-z (PMC6291945; doi:10.1186/s12864-018-5239-z)
Supplement: Supplementary file 3 — Figure S2: Distribution of genotyping accuracy of RNAseq data computed from a comparison with genotyping from a previously available SNP array [1] for the 12 individuals used in the study. Figure S3: Variation of the total SNP number and identical positions found with the chip data using 7 calling modalities times 3 options for missing values. Figure S4: Positions of SNPs discovered and genotyped with RNAseq across 12 Populus nigra individuals and along two genes. Figure S5: Graphical representation of the enrichment in GO terms (biological process) for the genes covered by at least 5 SNPs. (PDF 434 kb) [file 12864_2018_5239_MOESM3_ESM.pdf]

Additional file 3 for "Accuracy of RNAseq based SNP discovery and genotyping in *Populus nigra*"

Odile Rogier, Aurélien Chateigner, Souhila Amanzougarene, Marie-Claude Lesage-Descauses, Sandrine Balzergue, Véronique Brunaud, José Caius, Ludivine Soubigou-Taconnat, Véronique Jorge, Vincent Segura*

Correspondence: *vincent.segura@inra.fr

- **Supplementary figure 1:** Repetition quality control checked using genotyping from a previously available SNP chip (Faivre-Rampant *et al.* 2016, doi: 10.1111/1755-0998.12513).
- **Supplementary figure 2:** Distribution of genotyping accuracy of RNAseq data computed from a comparison with genotyping from a previously available SNP array (Faivre-Rampant *et al.* 2016, doi: 10.1111/1755-0998.12513) for the 12 individuals used in the study.
- **Supplementary figure 3:** Variation of the total SNP number and identical positions found with the chip data using 7 calling modalities times 3 options for missing values.).
- **Supplementary figure 4:** Positions of SNPs discovered and genotyped with RNAseq across 12 *Populus nigra* individuals and along two genes.
- **Supplementary figure 5:** Graphical representation of the enrichment in GO terms (biological process) for the genes covered by at least 5 SNPs.

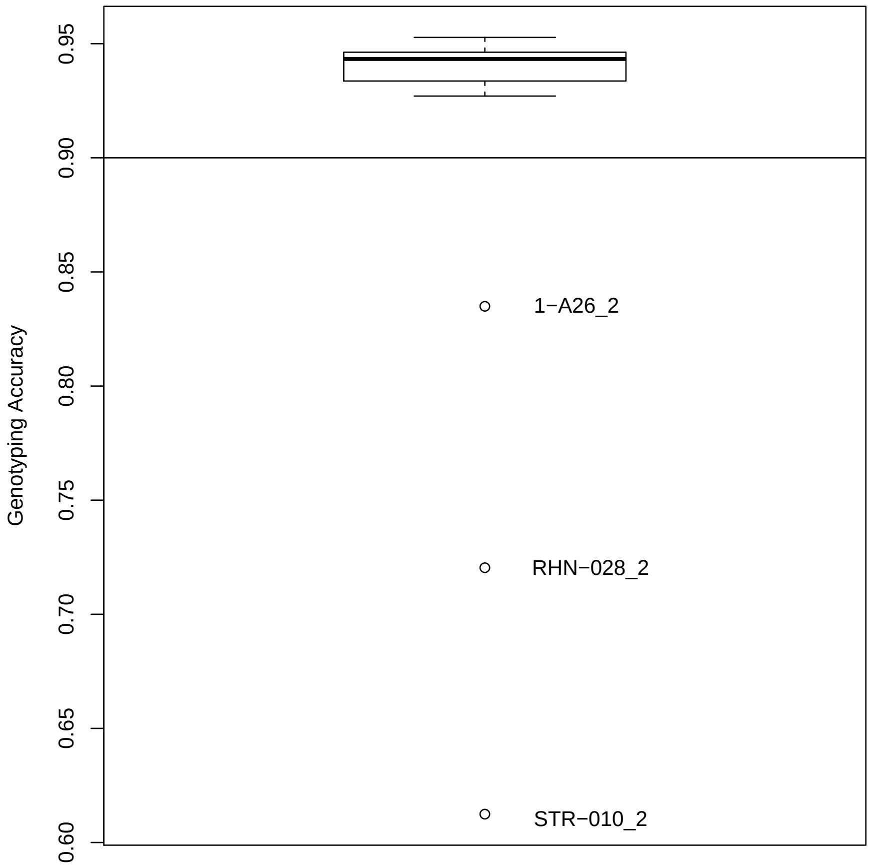

Supplementary Figure 1: Repetition quality control checked using genotyping from a previously available SNP chip (Faivre-Rampant *et al.* 2016, doi: 10.1111/1755-0998.12513). Boxplot of the genotyping accuracy across the 24 samples under study.

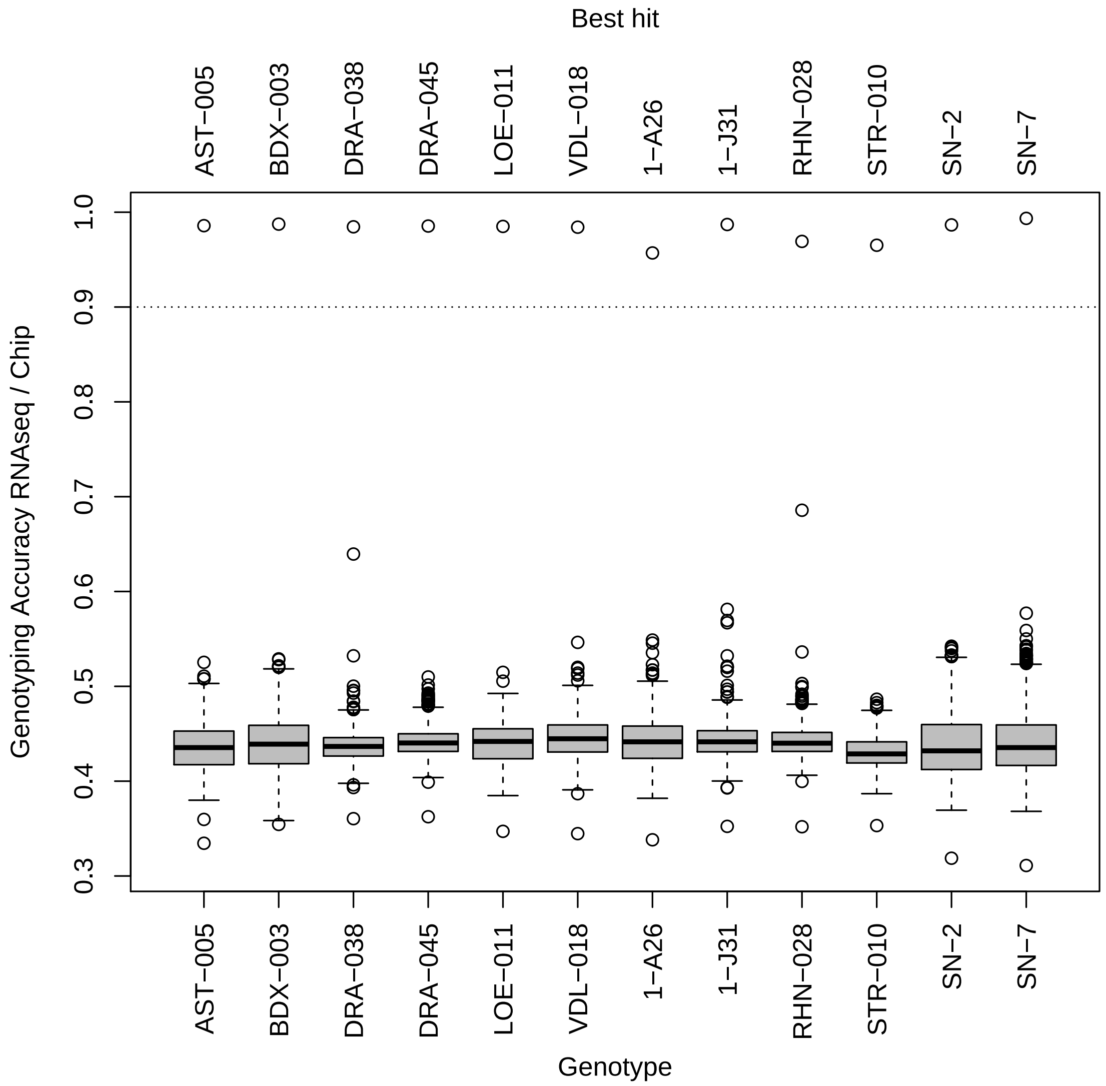

Supplementary Figure 2: Distribution of genotyping accuracy of RNAseq data computed from a comparison with genotyping from a previously available SNP array (Faivre-Rampant *et al.* 2016, doi: 10.1111/1755-0998.12513) for the 12 individuals used in the study.
Genotype of each individual of the RNAseq study (x axis) has been compared to all genotypes obtained with the SNP array, and genotyping accuracy has been calculated for each pair (y axis). The code on the upper part of the graph corresponds to the 'SNP array genotyped' individual giving the highest accuracy value.

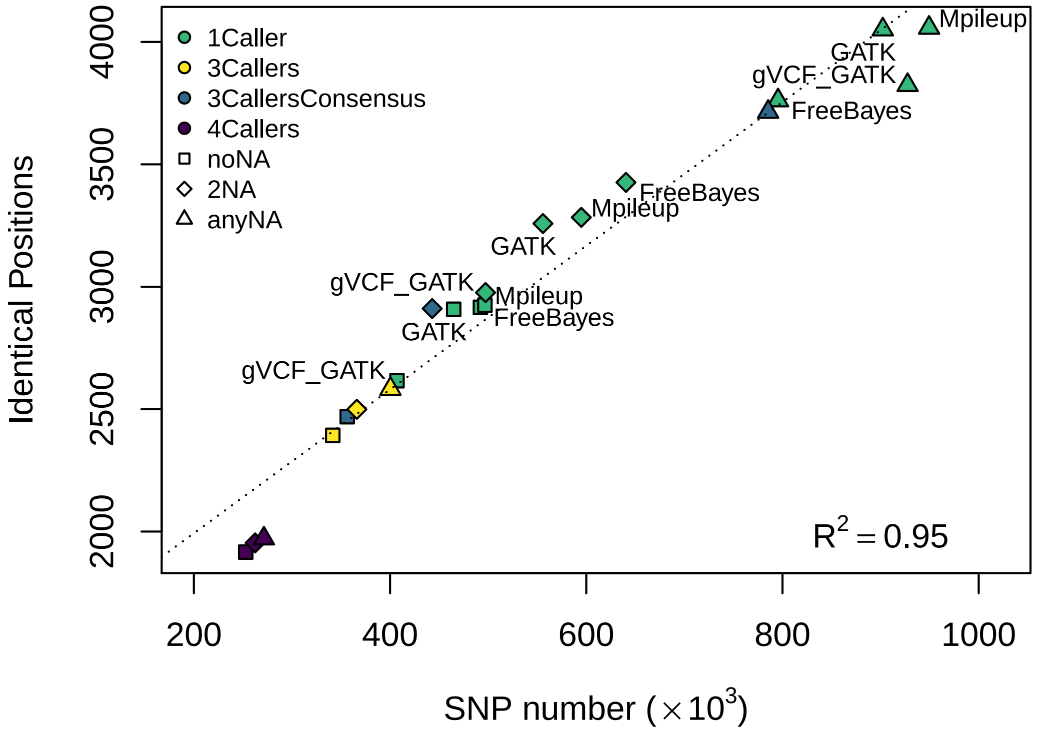

Supplementary Figure 3: Variation of the total SNP number and identical positions found with the chip data using 7 calling modalities times 3 options for missing values.
See Table 1 for the corresponding denominations.

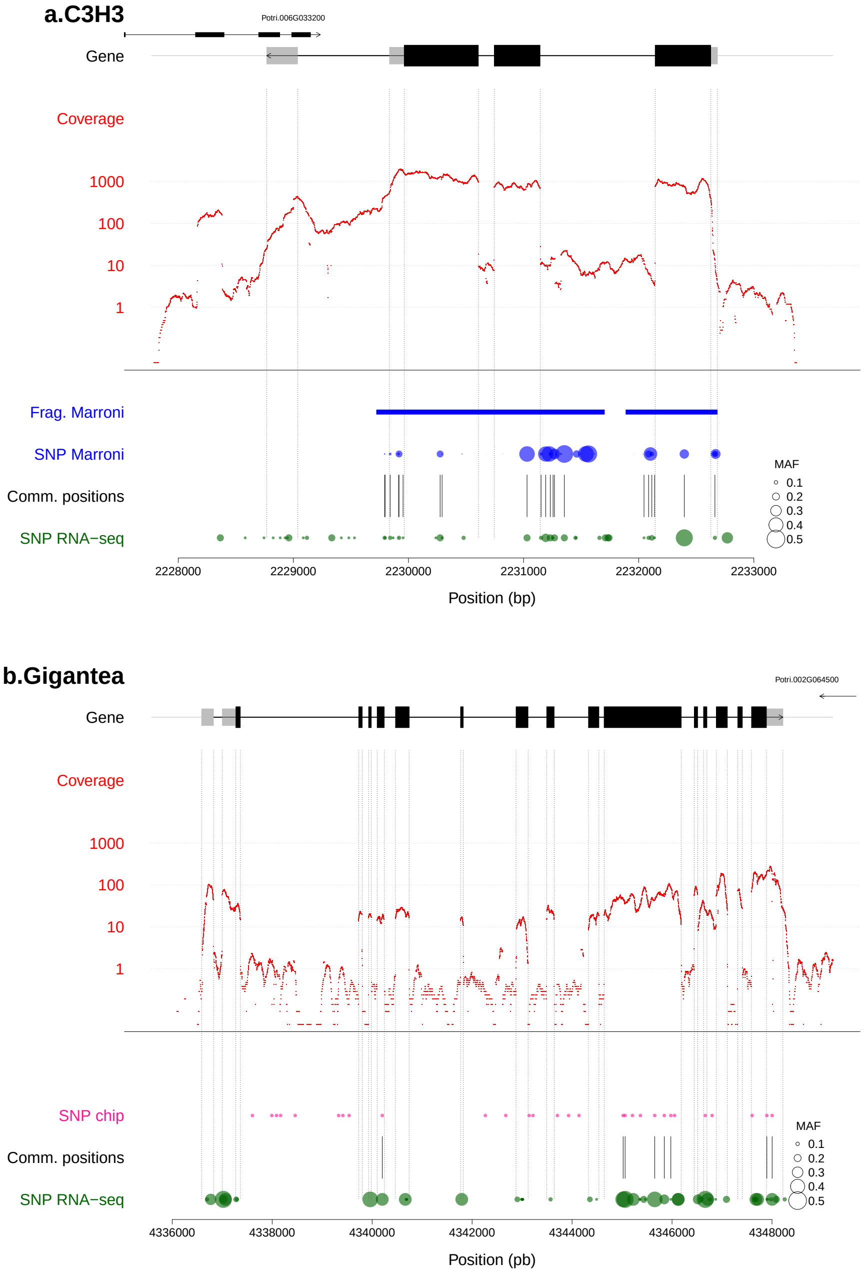

Supplementary Figure 4: Positions of SNPs discovered and genotyped with RNAseq across 12 *Populus nigra* individuals and along two genes.
a. "C3H3" (Potri.006G033300); b. "Gigantea" (Potri.002G064400). "Coverage" in red refers to the mean depth at each position among the 12 individuals; "Frag. Marroni" and "SNP Marroni" in blue refer to sequenced fragments and SNPs discovered and typed in Marroni *et al.* (2011a, doi: 10.1111/j.1365-313X.2011.04627.x & 2011b, doi: 10.1007/s11295-011-0391-5); "SNP chip" in pink refers to SNPs previously genotyped with a SNP array (Faivre-Rampant *et al.* 2016, doi: 10.1111/1755-0998.12513); "SNP RNA-seq" in green refers to SNPs discovered and typed in the present study with the modality "3CallersConsensus-noNA". Point symbol size for each SNP is proportional to its MAF across the 12 individuals.

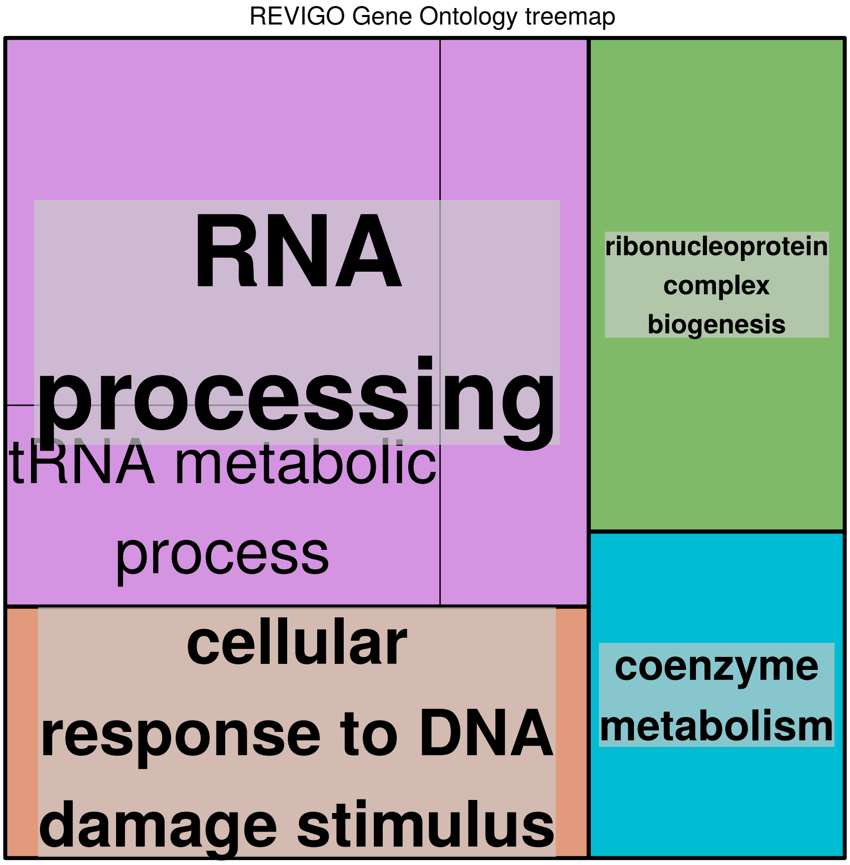

Supplementary Figure 5: Graphical representation of the enrichment in GO terms (biological process) for the genes covered by at least 5 SNPs. Enrichment and graphical representation were done with the R package topReviGO (Chateigner 2017, https://github.com/achateigner/topReviGO) on the 18,384 *Arabidopsis thaliana* orthologs to the genes covered by at least 5 SNPs in the present study.
